# Reflections on the transition to online teaching for health science education during the COVID-19 pandemic

**DOI:** 10.5116/ijme.610c.1580

**Published:** 2021-08-26

**Authors:** Nabeel Al-Yateem, Jacqueline M. Dias, Muhammad A. Subu, Mini Sarah Abraham, Fatma Abd El-baky, Amina AlMarzouqi, Syed Azizu Rahman, Ahmad Rajeh Saifan, Mohammad G. Mohammad, Intima Alrimawi, MoezAlIslam Faris

**Affiliations:** 1 Department of Nursing, College of Health Sciences, University of Sharjah, Sharjah, United Arab Emirates; 2 Department of Health Service Administration, College of Health Sciences, University of Sharjah, Sharjah, United Arab Emirates; 3 Applied Sciences Private University, Amman, Jordan; 4 Department of Medical laboratory Sciences, College of Health Sciences, University of Sharjah, Sharjah, United Arab Emirates; 5 School of Nursing and Health Professions, Trinity Washington University, Washington DC, USA; 6 Department of Clinical Nutrition and Dietetics, College of Health Sciences, University of Sharjah, Sharjah, United Arab Emirates

**Keywords:** Reflections, transition to online teaching, health science education, COVID-19 pandemic

## Introduction

In March 2020, COVID-19 declared a pandemic and major international public health concern.^1^ As of January 23, 2021, there were more than 96 million confirmed cases and over 2 million deaths worldwide; the United Arab Emirates (UAE) reported 267,258 confirmed cases and 766 deaths.^2^ Governments implemented interventions to mitigate the pandemic (e.g., social distancing, border closures, restrictions on local, national, and international movements), along with moving to online/distance work (where possible), closure of non-essential services/shops, and closure of daycare nurseries, schools, and universities.^3^ Worldwide, healthcare systems experienced increased pressure. Efforts to combat the pandemic included: designating COVID-19 hospitals; expanding acute/intensive care units ready; increasing the capacity of essential public health services; training, repurposing, and mobilizing the health workforce; and maintaining essential services while freeing up capacity for COVID-19 response.^4^ Healthcare professionals were also under pressure, working in high-risk, high-stress environments with personnel shortages, changing treatment protocols, new models of care, and critically ill and dying patients.^5^^-^^7^

The pandemic also impacted educational systems. Schools and universities were closed, and traditional education processes were shifted to online methods. Although many medical schools had plans for online basic and clinical medical education, COVID-19 challenged their implementation readiness. Complicating factors included underlying perceptions that clinical and health courses could not be taught online, lack of resources, and lack of preparation. Clinical and health sciences were further impacted because of the reliance on clinical practice in hospitals and other clinical settings, as medical and allied health sciences education depends on direct patient care in these settings and simulation laboratories.^8^ In many countries, clinical training and simulation were stopped and moved online. Clinical settings also canceled student training, especially for the foundational years.

This paper presents reflections on the transition to online learning in health sciences education in the UAE. We highlight issues encountered and solutions implemented to strengthen future responses to similar situations and improve current educational systems. Despite the UAE's rapid development (e.g., industry, tourism, healthcare, and education), many areas still need strengthening. The present paper seeks to help policymakers build more resilient and efficient systems and services. Similarities between the UAE and neighboring Gulf Cooperation Council and the Middle East/North African countries mean these challenges and solutions may resonate with these countries and support the implementation of effective online health sciences education. As the educational response to the pandemic was similar globally, the authors argue that many of the challenges faced by educators and students were also similar. Therefore, this reflection may seed discussions on how to move forward and strengthen the online education process.

### Implementing online learning in the UAE

The UAE Ministry of Education (MOE) moved education online in March 2020. Students had to resume their studies entirely through distance/online learning. Strategies implemented to ensure a successful transition to online learning included training for faculty, instructors, and administrators. The MOE also established operations support centers to monitor the distance learning process and ensure a smooth experience with sufficient information technology (IT) resources and effective communication between students, families, and instructors.^9^ The MOE coordinated with the Telecommunications Regulatory Authority and telecommunication providers to facilitate free mobile Internet packages for UAE families without a home Internet connection.^9^ The MOE also established acceptable/unacceptable student behaviors, and procedures for dealing with unacceptable behaviors.^9^ Training was implemented to prepare education institutions, academics, administrators, and students/families for online teaching and learning. However, this training was sudden and covered a large amount of content in a short time. In addition, most training were delivered online; trainees were required to use multiple Internet-based programs with which they were unfamiliar.

The complete switch to online learning was an accepted alternative in higher education, especially for entirely theoretical courses. However, it presented challenges for practical or clinical courses in health sciences education. To compensate for these challenges and the loss of skill acquisition, instructors implemented strategies such as online discussion of simulated clinical scenarios and software that simulated clinical skills (i.e., Med+safe 2D simulation for medication administration and critical thinking, Wiley Clinical Keys, and Elsevier clinical skills). However, instructors and students noted the problem of skills/knowledge acquisition persisted, and found the strategies used were less effective than expected. This was inconsistent with the evidence of the effectiveness of online teaching in health science education. A systematic review (59 studies: 6750 health sciences students) that evaluated the effectiveness of online learning compared with traditional or alternative learning methods concluded online learning was equivalent or superior to conventional learning and should be encouraged.^10^ Another systematic review (19 studies) explored whether an online or blended learning paradigm enhanced the teaching of clinical skills in undergraduate nursing.^11^ The findings suggested online learning was no less effective than traditional methods for teaching clinical skills, knowledge gain, and user satisfaction.^11^ Finally, a recent systematic review and meta-analysis (21 studies: 3684 health professionals and students) synthesized evidence regarding the efficacy of adaptive e-learning environments in improving knowledge, skills, and clinical behavior in health professionals and students.^12^ That review indicated online methods were effective, especially for learning skills rather than factual knowledge.^12^ However, most studies in those reviews were conducted in Western countries and did not consider teaching in pandemic conditions. In addition, they explored partial online or blended teaching rather than fully online programs. It was also clear that online and blended teaching was planned and organized, the academics were prepared to teach in this way, and the online teaching was part of the study plan with resources allocated in advance.

The effectiveness of online teaching as reported in the literature and the challenges encountered in the UAE raised questions and triggered reflections about the online teaching methods used. This reflection addresses the following key questions. What are the gaps in the implementation of online teaching? Why was online teaching not as effective as described in the literature? Have we adopted an effective online teaching strategy, or did we simply and unknowingly move traditional teaching online?

### Instructors' reflections

For instructors, online teaching was a sudden change, as face-to-face teaching was the norm for higher education in the UAE. Many UAE universities had not previously conducted any fully online courses. In most cases, online tools had only been used for exchanging assignments and course-related materials between instructors and students. During the pandemic, instructors had to suddenly move from their "normal reality" to a new situation where all teaching components (lectures, assignments, feedback, meetings, and assessments) were delivered online. Many instructors had never practiced online teaching and were unfamiliar with the associated principles, theories, and strategies; they had to learn how to manage these factors during the lockdown. Anecdotal evidence indicated lack of knowledge about the new medium meant many instructors felt insecure and overwhelmed. However, they supported each other to cope with the new processes, describing each day as an "expedition trying out various apps and software." Despite support from higher education institutions, the transition to online learning was initially perceived as chaotic. Although most institutions offered technical support for academics and students, there were insufficient technicians available to meet all requirements.

Moreover, the technicians were not always qualified to respond to this situation. In addition, academics were accustomed to managing the teaching process, including exams and quizzes. Needing help and support in these areas made them uncomfortable and anxious.

Problems experienced by educators included starting a class using one technology but needing to move to another platform because of technical difficulties. Many did not know how to resolve technical glitches and "hopped" from one medium to another. In addition, instructors felt disadvantaged as they did not know how to make the class interactive, conduct labs/group activities, or conduct team-based learning or focus group discussions using the available technology. This caused stress and dissatisfaction. Another challenge for instructors was talking to a blank screen because students were not expected to open their videos for cultural reasons. Sometimes, instructors would ask questions only to find that few students were active or present. In addition, some students were dominating and vocal whereas others were quiet or not present at all, as they had left their computer connected but unattended. It was also noted that overall student participation was low. Some instructors were able to encourage student engagement through small assignments, quizzes, and interactive class sessions where students had to work together to solve problems. However, many instructors were not skilled in encouraging student engagement and reported feeling their role was merely "a deliverer of content." Using the chatbox function was considered helpful because instructors felt they were heard and that there were students "on the other side of the screen." Online teaching also required additional roles (e.g., technical, managerial, and new social roles). Instructors had to motivate students, develop rapport, make them feel welcome and attended, and place them at the center of the learning process rather than the course content. Instructors also had to help students socialize for group work, especially if they had not met each other before. In such cases, they reported it was fulfilling to see a learning community evolving. It appeared that students enjoyed collaborative learning and freedom from the teacher's involvement.

An important challenge that emerged was clinical training, particularly how it could be replicated online. The simulation was not possible as physical attendance at the university was forbidden. Online scenario discussions, online videos, live demonstrations, and skills-simulating software were explored and implemented appropriately. As the lockdown eased, live laboratory sessions were introduced with necessary precautions and social distancing. During the pandemic, many senior-level students were sent to low-risk practice areas if their involvement was possible. However, the teaching of clinical skills remained the most affected part of students' study. This caused major concern as skills acquisition is essential for health sciences education. Academics also faced problems when assessing students because assessments in the UAE usually used objective evaluation methods (e.g., multiple-choice questions). However, evaluation methods used for the online evaluation included open book exams, assignments, and open-ended questions. Implementing these evaluation methods was challenging because students were unfamiliar with these methods. Most instructors ensured they were accessible and available if students had concerns or issues, but actual accessibility varied among instructors. Checking student performance and continuous communication with students regarding their health and studies is important but was perceived as frequently missing and needing improvement.

After almost one year of experiencing online teaching, instructors reported adaptation of their skills and improved confidence and satisfaction in delivering online teaching. They had learned to navigate and manage online platforms and taught students how to access learning material, submit assignments, take quizzes and exams, and be active on discussion boards. Many instructors perceived online teaching as better as a group activity as breakout groups offered a way for students to interact with each other and give feedback. Some instructors developed the knowledge and skills to practice collaborative learning and appreciate students' social presence (although virtual). However, clinical training and skill acquisition remained challenging, with continued restrictions on clinical training in hospitals and other clinical settings. The immature simulation infrastructure in many UAE higher education institutions was also an issue, as it increased the reliance on training in actual clinical sites. Key issues for simulation were the limited human resources/capability and the lack of trained or specialized simulation instructors. In addition, despite the desire and equipment to implement these strategies, the actual implementation was not ideal or even impossible in many institutions.

### Students' reflections

Students appreciated that they did not need to commute to and from campus physically. They were also grateful that they could stay safe from infection at home while continuing their studies online. Many students welcomed the move to online teaching to balance their work, home, children, and studies. However, various pros and cons of the situation emerged. Online teaching was initially delivered using a learning management platform that students had been introduced to when they registered for their classes. The only difference in this platform in the full online switch was videoconferencing and collaboration facilities that allowed virtual classrooms. Students who were not technically skilled or did not have the necessary technology and Internet connection at home had difficulties. Using other social networking platforms (e.g., WhatsApp) with the whole class enabled students to help each other. Technical challenges related to the Internet infrastructure also emerged. Some students found it hard to deal with the technology needed for online learning, especially as classes were sometimes delayed because of poor Wi-Fi connections. Some students required constant help from IT staff or other faculty members. Students also reported feeling they did not have to participate in class; not having a teacher physically present made them less attentive. Over time, this negatively affected their grades, and students reported realizing they would have to take ownership of their learning and be self-motivated to study. Students reported that the lack of interaction with their instructors and classmates created a huge void. Teamwork in the online environment was considered much harder, and students felt "disjointed." They reported using a keyboard to chat with their professor was not the same as talking with them in person, and sometimes became an impediment because of language. Although a microphone was helpful, it was also associated with problems such as microphone disturbance, background noise, and voice lagging.

An important point raised by students was that the use of traditional methods (e.g., PowerPoint) to teach online classes was problematic. For example, being taught via PowerPoint meant students had to look at the screen and follow the presentation while also following the teacher in a small screen at the bottom of the presentation. This was perceived as a poor combination; therefore, online teaching may need to be implemented using more compatible teaching methods. Furthermore, students reported that the volume of course work, quizzes, and exams had stayed the same or increased compared with before going online. Students questioned whether this was compatible with online teaching, which depends on flexible, self-directed learning and less traditional assessment methods. Some students also felt that they missed learning and using valuable skills (e.g., general presentation and public speaking skills, important clinical skills). Exam stress was another issue. Taking examinations online was perceived as having advantages and disadvantages. For example, being in an examination hall had a more formal, serious tone than taking that exam at home. Many students indicated they felt weak and anxious on top of general exam stress. In addition, some students lived in homes with many people, few rooms, or other disruptions, meaning taking exams was distracting. However, they noted that driving to and from university for a 2-hour exam and the stress of entering an exam room was eliminated. Strategies used to support online exams included decreasing the exam time and using short-answer questions. However, some students noted that this added more pressure as they preferred to use the whole exam time to answer the questions.

### Development of online teaching

The rapid advancement of technology, the Internet, and communication methods mean online learning is offered by many institutions around the world, including prestigious institutions. There is strong evidence for the effectiveness of this method. Different online learning methods have been explored and compared, such as the use of mobile communication technologies, synchronous versus asynchronous online courses, massive open online courses, and personal learning environments.^13^^-^^18^ The UAE had also started integrating technology into education.^19^ However, some universities lagged in terms of offering of online courses, which were limited to modules in onsite courses, short courses, or lectures. However, the COVID-19 pandemic meant online courses were offered in most subjects, and complete programs and courses were also delivered online.

### Tenets of online teaching

Successful online teaching depends on flexible timetables that suit different learner lifestyles, making it a viable alternative to on-campus study.^20^^, ^^21^ This flexibility is a major benefit of online learning, but also means students must independently manage their own learning time. Facilitators or instructors should encourage student autonomy and engagement by establishing different learning pathways and providing a variety of materials so students can make choices aligned with their interests. Instructors also have to teach and encourage time management.^20^^, ^^21^ Institutional support for learners is another important consideration. Online learning requires self-motivation, and educational support is essential. For example, tutor feedback is an important component, and tutors should ensure that online students receive the same level of support that they would receive on campus.^20^^-^^22^ Effective online teaching requires instructors to connect with students, form relationships, and be present on the online platform. This community aspect is a valuable commodity that is often overlooked. Students want their facilitators to support them, be active in their course, and create a sense of belonging.^22^ Instructors should not underestimate the impact of authentically relating to online students, especially as online learning can make learners feel isolated and stressed.^20^^-^^22^ Many students also have family/work responsibilities or are experiencing crises or stress (e.g., a life-threatening pandemic) while completing their education. Effective online teaching, therefore, requires instructors to have a presence, empathy, awareness, and actively connect with students.^20^^-^^22^ This engagement means more announcements, videos, or emails. It requires responding to student communication and making their progress an important target. This builds a strong perception of individualized care, which is valued by students.

Instructors should connect with students using various methods, including using smartphones or computers to create informal videos in their own space. It is also important for instructors to allow time for informal communication with students about stress; instructors need to treat students as individuals who need compassion. For example, instructors could share experiences from their own life and work with students, such as difficult deadlines or other issues. An important role for online instructors is to creating virtual classroom communities as an alternative to the traditional physical classroom communities. Physical learning classrooms foster connection and motivate interaction and enhance engagement. In online teaching, instructors need to create similar connections using virtual communities, thereby improving their teaching and students' experiences. As well as clear learning objectives, content, and assessments, students need instructors that are caring and partner with them, especially in their struggles.^20^^-^^22^ Effective online teaching also depends on the careful determination of the course content, pedagogy, and assessment.^21^^,^^23^ Teaching online differs from physical teaching. Instructors should plan a mix of asynchronous and synchronous activities as relevant to the course, including video, non-video, and peer learning communities; lectures; and case-based small group discussion.^21^^, ^^23^ Finally, thoughtful planning for both asynchronous and synchronous instruction is needed on when and how to use or record video, engage directly with students and build communities, and assess and gauge students' understanding.^21^^, ^^23^

### Conclusions and recommendations

This reflection activity may inform future improvement initiatives. The authors argue that as the educational response to the pandemic was similar regionally and globally, many of the challenges faced by educators and students are also similar, making this reflection useful in both local international contexts. Online teaching must be designed thoughtfully based on the principles of online methods and conducted in a skilled manner. Moving classical direct lecturing to an online medium does not constitute proper implementation of online teaching and is unlikely to produce the desired learning outcomes. To be effective, online teaching requires input from different parties, including supportive educators who are skilled in online teaching, students who are attentive, responsible, and looking to learn, appropriate IT infrastructure, and IT personnel to support instructors and students and resolve technical difficulties. Further, online teaching experts and content designers are needed to assist instructors to design and conduct their courses. Compared with traditional face-to-face teaching, online teaching requires extra effort from educators, who are expected to be available for students, supportive, and proactive in initiating and maintaining interactive online communities. This raises two issues. First, not all instructors can or are ready to do this. Those who do not have the required capacity or skills should be supported during this process. Second, instructors should be allowed adequate time to perfect the online teaching process; they should not be overburdened with other tasks that distract them from their online presence and duties.

The sudden move to online teaching presented an opportunity for the UAE. The higher education community was initially skeptical about this teaching method, which was considered substandard and not equivalent to the standard traditional teaching. The move to online learning during the pandemic showed that many courses and programs could be effectively delivered online. This process should now be integrated into mainstream education, and a blended method of teaching should be encouraged. This will allow working students to take online classes instead of face-to-face classes. Instructors will also be prepared for online teaching should another switch be enforced by a similar situation. In addition, learning will be optimized, especially as online learning was beneficial for some courses and subjects. Finally, there are financial benefits for both students and institutions. Online teaching and learning can be less costly for both students and institutions. It will also allow students to study while working and mean institutions can direct funds and resources that were used for face-to-face teaching to other important purposes.

### Conflicts of Interest

The authors declare that they have no conflict of interest.
